# Educational qualification differences and early labor market exit among men: the contribution of labor market marginalization measured across the working life

**DOI:** 10.1186/s12889-022-13397-1

**Published:** 2022-05-20

**Authors:** Emelie Thern, Daniel Falkstedt, Melody Almroth, Katarina Kjellberg, Jonas Landberg, Theo Bodin, Bo Melin, Tomas Hemmingsson

**Affiliations:** 1 Department of Public Health Sciences, Stockholm University, Stockholm, Sweden; 2 Institute of Environmental Medicine, Karolinska Institute, Stockholm, Sweden; 3 Centre for Occupational and Environmental Medicine, Region Stockholm, Stockholm, Sweden; 4 Department of Clinical Neuroscience, Karolinska Institute, Stockholm, Sweden

**Keywords:** Early exit, Voluntary exit, Involuntary exit, Educational inequalities, Labor market marginalization

## Abstract

**Background:**

The present study aims to investigate the association between educational qualification and early labor market exit among men and to examine the contribution of labor market marginalization measured across the working life on this association.

**Method:**

A register-linked cohort study was conducted including men who completed military service in 1969/70 (born between 1949 and 1951) and were alive at age 55 and not disability pension beneficiaries (*n* = 40 761). Information on the highest level of educational qualification and the outcome of early exit (disability pension, sickness absence, unemployment, and early old-age pension) was obtained from Swedish nationwide registers between the ages of 55 and 64 years. Labor market marginalization was defined as periods of long-term unemployment and sickness absence over the working life and up to follow-up. Cox regression analyses were used to obtain hazard ratios (HR) with 95% confidence intervals (CI).

**Results:**

Low-educated men were more likely to leave the labor force early due to disability pension or sickness absence (HR: 2.48), unemployment (HR: 2.09), and early old-age pension with- (HR:1.25) and without -income (HR: 1.58). Labor market marginalization across the working life explained a large part of the association for the more involuntary early exit routes (disability pensions, sickness absence, unemployment) and explained very little with regards to the more voluntary early exit routes (early old-age pension with and without income).

**Conclusion:**

Exposure to labor market marginalization across the working life was important in explaining educational differences in early labor market exit due to disability pension or sickness absence and unemployment. This study underscores the importance of identifying and implementing preventive measures in the workplace (e.g. adaptions) to prevent new spells of sickness absence and unemployment, especially among low educated individuals.

**Supplementary Information:**

The online version contains supplementary material available at 10.1186/s12889-022-13397-1.

## Background

In the past few decades, Sweden and many other EU member states have undergone substantial pension reforms centered on the introduction of higher pensionable ages, tighter eligibility conditions for disability pensions, and reductions in early-retirement opportunities to counteract the financial consequences of an increasingly older population [1]. However, prolonging working life may be challenging, especially for individuals with lower levels of education [2]. Evidence suggests a large proportion of workers, especially individuals with lower levels of education, leave the workforce early and permanently due to health, work circumstances, and social- and economic factors [2–8]. Previously, disability pension was virtually the only early exit route out of the labor force in Sweden. However, given the tighter eligibility criteria for disability pension introduced, the use of alternative exit routes (e.g. unemployment, sickness absence, early old-age pension) from the labor market have increased as previously seen when a change in the social system occurs [9–12].

Within the research field, the push–pull model refers to determinants influencing older workers’ early exit from the labor market [3]. The basis of this model is that individuals are either pushed (involuntary) or pulled (voluntarily) out of the workforce. Disability pension, sickness absence, and unemployment could be considered involuntary exit routes, as the individuals using one of these exit routes are most likely pushed out of the labor force early. For other individuals, retirement might be perceived as more attractive than being employed and consequently, they choose a more voluntary early exit route and take out their old-age pension before the statutory pension age [3]. Leaving the labor force on a voluntary or involuntary basis shows different associations with later health and well-being; individuals leaving involuntary appear much worse off with regards to health and well-being later in life [13]. Individuals with a lower level of education are at an increased risk of involuntary exit routes [2]. With regards to more voluntary exit routes, the underlying reasons and motives for taking out old-age pension early could differ between individuals with high and low levels of education. On the one hand, individuals with lower levels of education might need to take out their old-age pension early as they might not be eligible for a disability pension or sickness absence despite having health problems [14]. On the other hand, individuals with higher levels of education might choose to voluntarily leave the labor market early as they have the finical resources to do so [15], which suggests that the well-established educational differences in disability pension might also exist in other exit routes [2, 16–22].

Previous research on factors potentially explaining educational differences in early exit has mainly focused on a single exit route (i.e. disability pension) or used a comprehensive definition of the outcome [17, 19–22]. The few studies that have been able to differentiate the different exit routes have found that the educational differences in disability pension, sickness absence, unemployment, and early retirement, were partly explained by health, lifestyle, work histories, and work characteristics [23–25]. Given that early life experiences are systematically linked to outcomes later in life, it is important to study early exposures and transitions over the life course, as opposed to examining exposures at a single point in time [26]. The few studies that have investigated the effect of factors measured before labor market entry have found that early factors related to lifestyle, health, personality, and especially cognitive ability explained a large part of the association between education and disability pension [17, 18]. These studies were also based on the same conscription cohort as in the current study, as this important information is unfortunately only available for men that have undergone conscription examination [17, 18]. Evidence has also shown that poor health, which is more prevalent among lower educated individuals, is a crucial and strong predictor of an early exit, especially concerning disability pension [4, 7, 19, 20, 24]. A recent study found that differences in ill-health explained a large part but not all of the association between education and disability pension, and education and unemployment [7].

Less is, however, known about the effect of labor market marginalization across the working life on the educational differences in an early exit. Individuals exposed to long-term unemployment or long-term sickness absence could be considered to be marginalized from the labor market as they have a low attachment to the labor force but are expected to return to the labor market at a later stage. Previous research on work-life expectancy has found that individuals with lower levels of education tend to experience more unemployment spells, as well as more and longer spells of sickness absence during their working life compared to individuals with higher levels of education [8, 18, 25]. This is of importance as being marginalized from the labor force can result in lower salaries, recurring unemployment, and sickness absence, as well as an early exit from the labor force [27–30]. Considering that employment histories have become more de-standardized and more fragmented today than they have previously been [31–33], it is of great importance to gain a better understanding of the contribution of labor market marginalization across the working life in explaining the potential educational differences in an early exit.

The present study aims to investigate the association between educational qualification and early labor market exit among men and to examine the contribution of labor market marginalization measured across the working life on this association. As previous research has highlighted the importance of taking early life circumstances into account we will follow a large cohort of men from childhood up to the age of 64 years old.

## Methods

### Study population

**Fig. 1:**
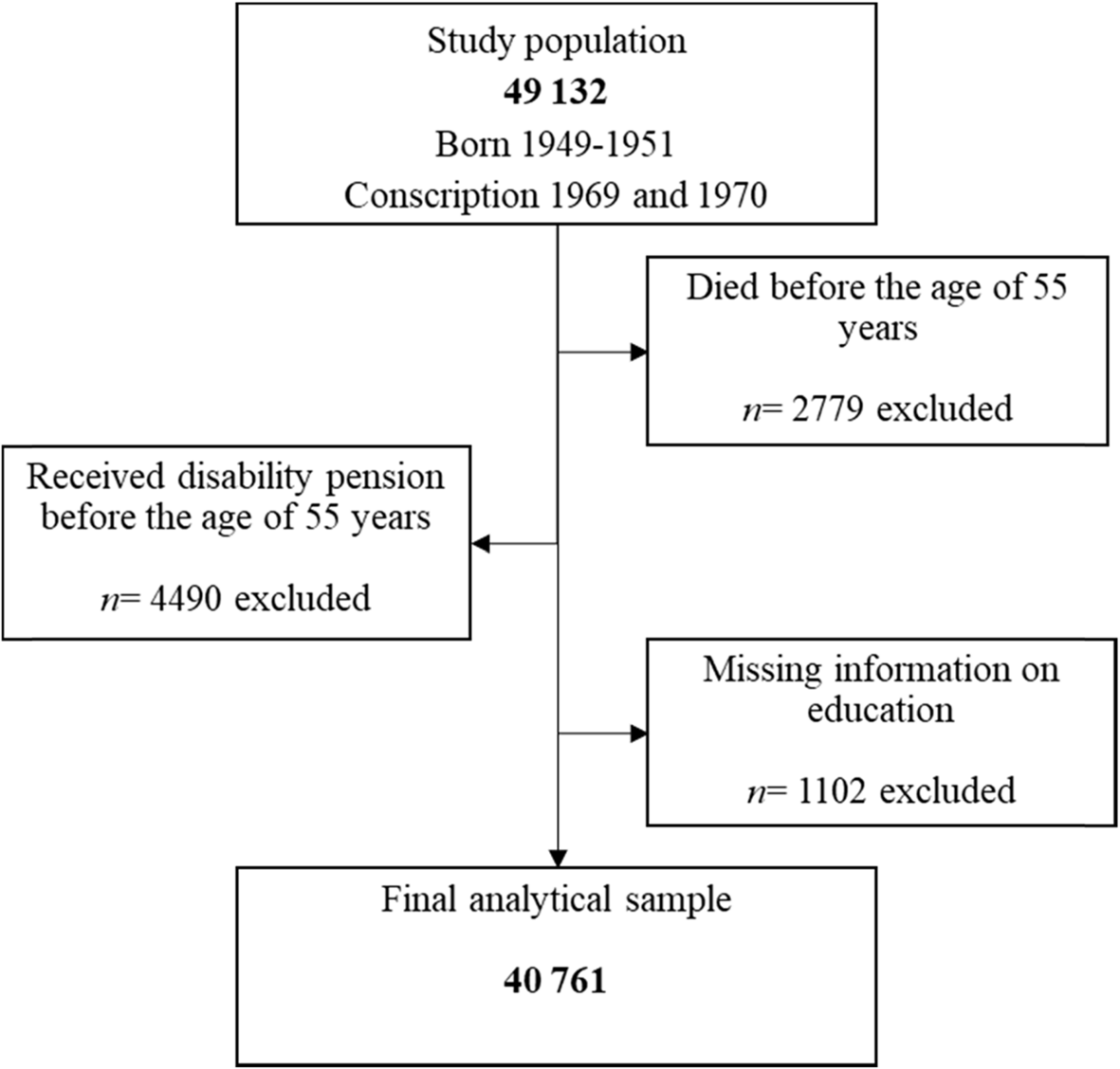
Flow chart describing the selection process of the participants

A register-based longitudinal study based on the Swedish 1969/70 conscription cohort was conducted. The conscription cohort includes all men born between 1949 and 1951, who completed military service in 1969/1970 in Sweden (*n* = 49 132). Military service was obligatory for all males aged 18–20 years in Sweden at this time, consequently, only 2 to 3% of the general population were exempted from conscription due to severe handicaps or congenital disorders. The present study is based on all men who were registered in Sweden (between 2004 and 2006) and alive at the age of 55 years. Individuals that received disability pensions before the age of 55 were excluded, as well as individuals with missing information on their level of education. Excluded individuals were generally worse off during childhood and late adolescence, as well as had experienced more labor market marginalization, both in terms of long-term unemployment and long-term sickness absence, across their working life compared to the included men (Supplementary Table 1). The final analytical sample consisted of 40 761 men (Fig. 1).

### Exposure: educational qualification

Information on the highest level of educational attainment was collected from the Longitudinal Register of Education and Labour Market Statistics (LISA) the year the men turned 55 years (2004–2006). The LISA register was established in 1990, updated yearly by Statistics Sweden, and contains information on various factors related to education, income, and the labor market for all citizens from the age of 16 years [34]. The exposure variable was categorized into five separate groups based on the number of years in education: ≤ 9 years (primary), 10–11 years (2 years of upper secondary school), 12 years (3 years of upper secondary), 13–14 years (2 years of university), and ≥ 15 years (3 or more years of university), with the last group serving as the reference category.

### Outcome: Early Exit

The outcome of Early Exit was defined using five different exit routes: disability pension, long-term sickness absence, long-term unemployment, old age pension with income, and old age pension without income. Information on the outcome of disability pensions, sickness absence, and unemployment were collected from the LISA register from the year the men turned 55 years old (2004/2005/2006) until the year the men turned 64 years old (2013/2014/2015). For the outcome of early old-age pension, the information as collected from the year when the men turned 60 years old (2009/2010/2011) since old-age pension can earliest be taken out at the from the age of 61/62 years. In these analyses, those men who died or received disability pensions between the age of 55 and 60 were also excluded (*n* = 868).

In line with previous research, disability pension was defined as being granted full- or part-time disability pension during follow-up [18, 35]. Long-term sickness absence was defined as receiving sickness benefits from the Swedish Social Insurance Agency for 90 days or more annually [18, 35]. Similarly, long-term unemployment was defined as being registered as unemployed for at least 180 days in one year [18, 35]. In Sweden, old-age pensions can be taken out from the age of 61/62 years. Men that began taking out their pension before turning 65 years old and who continued to have an income above one Price Base Amount (PBA) the following year were considered as being early old-age pensioners with income, whereas individuals taking their pension out early with an income below one PBA the following year was considered as early old-age pensions without income [36]. The cut-off was set at 65 years old as this is the norm for retirement in Sweden [37].

In the main analyses, disability pension and long-term sickness absence were merged into one outcome- ‘health-related early exit’—since the majority of disability pensioners received sickness absence benefits before being granted disability pension. In the current study sample, more than 80% of the individuals who received disability pension had registered sickness absence during the follow-up period.

### Labour market marginalization

Labor market marginalization was defined as periods of long-term unemployment and sickness absence over the working life and up to the start of follow-up. Adapted from previous research in our group, four different measures of unemployment were included; youth unemployment, unemployed in young adulthood, unemployed in middle adulthood, and unemployed in older adulthood [18]. Information on previous unemployment was collected from three different sources; the conscription examination, Swedish Income, and Tax Register, and the LISA register. From the conscription examination information on youth unemployment, defined as experiencing more than three months of unemployment before the age of 18 years, was obtained. Similar to previous research [38], information from the Swedish Income and Tax Register on receiving unemployment benefits (Unemployment Insurance Funds) or unemployment assistance (Social Insurance Agency) was obtained between 1974 and 1991. Individuals receiving any unemployment benefit or assistance (regardless of the number of days in unemployment) for at least 4 out of the 15 years were classified as being unemployed in young adulthood. Using the LISA register and the same definition as the outcome, information on prior long-term unemployment in middle adulthood (between the age of 43/44/45 years and 49 years) and older adulthood (between the age of 50 years and 54 years or 59 years depending on the outcome) was included. Participants were considered to have experienced unemployment in middle adulthood and older adulthood if this was reported at least once during the specified time periods. Using the LISA register and the same definition as for the outcome, information on prior long-term sickness absence in middle adulthood was collected from 1994 to the year the men turned 49 years and prior long-term sickness absence in older adulthood was collected between the ages of 50 and 54 years or 59 years depending on the outcome. Participants were considered to have experienced prior long-term sickness absence in middle adulthood and older adulthood if this was reported at least once during the specified time periods.

### Other potential explanatory factors

The selection and inclusion of other important explanatory factors were selected based on previous research [17, 18]. Childhood SEP was defined using their fathers’ occupational class and was collected from the Swedish Population and Housing Census in 1960 when the men were 9–11 years old. Childhood SEP was categorized into seven groups according to the Swedish socioeconomic classification of occupations: (i) unskilled workers, (ii) skilled workers, (iii) low-level non-manual employees, (iv) intermediate non-manual employees, (v) high-level non-manual employees, (vi) farmers, and (vii) those not classified into a socioeconomic group [39].

In Sweden, all enlistees need to complete a standard medical assessment of their physical and mental health, as well as two survey questionnaires. From the conscription examination information on cognitive ability, health behaviors, and emotional control were obtained. The IQ test was based on four subtests designed to capture logical, spatial, verbal, and technical abilities [40]. From the four subtests, a global IQ score was calculated and standardized to give a Gaussian-distributed score between 1 and 9, with higher values indicating greater intellectual capacity. Health behaviors (i.e. smoking, alcohol, and BMI) were defined in line with previous research [17, 18]. Smokers were defined as smoking five cigarettes per day or more. Either consuming 250 g of 100% alcohol per week or more, have been detained because of drunkenness, using alcohol as an eye-opener, or being drunk often was considered risky use of alcohol. BMI was calculated using height and weight (weight/height^2^); having a BMI over 25 was considered as being overweight. Low emotional control (rated by a psychologist) was defined as having low-stress tolerance and/or anxiety, uncontrollable nervousness, anxiousness, or aggression, and reduced functioning due to psychosomatic symptoms.

From the conscription examination information on mental, and physical health was also obtained and included as a measure of health. A physician examined all men and, if considered necessary, a psychiatrist. Psychiatric and musculoskeletal diagnoses were defined according to the International Classification of Diseases version 8 (ICD-8); codes 290–315 and 710–738, respectively. From the National Hospital Discharge Register information on psychiatric diagnoses was obtained between 1973 and 2008/2009/2010 (i.e. between the ages of 22–24 years and 59 years). Psychiatric diagnoses were defined by the following discharge diagnostic codes: ICD-8 and ICD-9: 290–315; ICD-10: F00-F99.

### Statistical analysis

Pearson’s chi-square tests (χ^2^) were used to test for differences in baseline characteristics of the study population. The associations between the level of education and four different early exit routes were estimated by Cox proportional hazard models to obtain hazard ratios (HR) with 95% confidence intervals. The proportional hazards assumption was found not to be violated (all p-values ≥ 0.05) when using the test of Schoenfeld residuals. Information on the outcomes was obtained from registers that are updated annually; consequently, there was no exact date available for when the disability pension, sickness benefits, unemployment benefits, or old-age pension was received. Therefore, in line with previous research, the day and month of the outcome were set on the 2^nd^ of July as this is the middle of the year [17, 18]. For the outcome of health-related early exit and unemployment, person-time, in years, was counted from age 55 years (1 January 2004, at the earliest) until the date of an early exit, date of death, or until age 64 years (31 December 2015, at the latest), whichever came first. For the outcome of early old-age pension with and without income; person-time was counted from age 60 years (1 January 2009, at the earliest) instead since old-age pension can be taken out at the earliest from age 61/62 years in Sweden.

In the main analyses, the various measures of labor market marginalization across the working life were included individually as well as grouped together. In the full model all potential explanatory factors were included simultaneously; childhood SEP, cognitive ability, smoking, alcohol, BMI, low emotional control, mental and physical health, labor market marginalization across the working life.

The contribution of explanatory factors in explaining the education–early exit association was determined by the percent attenuation in the hazard ratio for education after the inclusion of the risk factor in question. The percentage of HR reduction was calculated as ((HR_crude_-HR_adjusted_)/(HR_crude_-1)) *100.

To examine how much of the marginalization effects are explained by individual background characteristics, additional analyses were conducted adjusting for labor market marginalization after initial adjustments of factors measured at conscripted and health-related factors had been entered into the model. Missing values on covariates were coded as separate categories, as similar results were obtained in the analyses of the complete cases (excluding individuals with missing information on potential explanatory factors) (Supplementary Table 2). All analyses were computed using Stata Statistical Software: Release 17.

## Results

### Baseline characteristics

Generally, men with lower levels of education had lower childhood SEP, lower IQ, more unhealthy behaviors during late adolescence, and worse health compared to men with higher levels of education (Table 1). Furthermore, these men also experienced more labor market marginalization, both in terms of long-term unemployment and long-term sickness absence, across their working life compared to men with higher levels of education.

**Table 1:** Baseline characteristics of study population, stratified by years of education

	≥ 15	13–14	12	10–11	≤ 9	*p*-value
	n(%)	n(%)	n(%)	n(%)	n(%)	
Total	7313 (17.9)	6063 (14.9)	6583 (16.2)	11 625 (28.5)	9177 (22.5)	
Childhood SEP^a^						
Unskilled worker	1295 (17.7)	1567 (25.9)	1988 (30.2)	4464 (38.4)	3904 (42.5)	< 0.001
Skilled worker	1025 (14.0)	1242 (20.5)	1465 (22.3)	2889 (24.9)	2083 (22.7)	
Low-level non-manual employee	1088 (14.9)	809 (13.3)	786 (11.9)	1006 (8.7)	562 (6.1)	
Intermediate non-manual employee	2249 (30.8)	1345 (22.2)	1254 (19.1)	1407 (12.1)	705 (7.7)	
High-level non-manual employee	1056 (14.4)	342 (5.6)	352 (5.6)	243 (2.1)	168 (1.8)	
Farmer	472 (6.5)	657 (10.8)	638 (9.7)	1333 (11.5)	1550 (16.9)	
Not classified	128 (1.8)	101 (1.7)	100 (1.5)	283 (2.4)	206 (2.2)	
IQ^b^						
High (7–9)	5000 (68.4)	3345 (55.2)	2495 (37.9)	2021 (17.4)	830 (9.0)	< 0.001
Medium (4–6)	2180 (29.8)	2501 (41.3)	3466 (52.7)	7062 (60.8)	4916 (53.6)	
Low (1–3)	129 (1.8)	214 (3.5)	618 (9.4)	2531 (21.8)	3421 (37.3)	
Missing	4 (0.1)	3 (0.1)	4 (0.01)	11 (0.1)	10 (0.1)	
Health behaviors^b^						
Smoking ≥ 5 cigarettes/day	2071 (28.3)	2147 (35.4)	2880 (43.8)	6055 (52.1)	5087 (55.4)	< 0.001
Risky use of alcohol	1017 (13.9)	912 (15.0)	1200 (18.2)	2751 (23.7)	2267 (24.7)	< 0.001
BMI ≥ 25	258 (3.5)	307 (5.1)	381 (5.8)	794 (6.8)	766 (8.4)	< 0.001
Low emotional control^b^	1747 (23.9)	1408 (23.2)	1604 (24.4)	3441 (29.6)	3121 (34.0)	< 0.001
Psychiatric diagnosis^b^	571 (7.8)	418 (6.9)	519 (7.9)	1324 (11.4)	1245 (13.6)	< 0.001
Musculoskeletal diagnosis^b^	1224 (16.7)	984 (16.2)	988 (15.0)	1903 (15.0)	1607 (17.5)	< 0.001
Inpatient-care psychiatric diagnosis^c^	178 (2.4)	198 (3.3)	239 (3.6)	614 (5.3)	521 (5.7)	< 0.001
Employment histories						
Youth unemployment^b^	206 (2.8)	250 (4.1)	411 (6.2)	1766 (15.2)	1835 (20.0)	< 0.001
Unemployed in young adulthood^d^	219 (3.0)	237 (3.9)	244 (3.7)	988 (8.6)	471 (5.1)	< 0.001
Unemployed in middle adulthood^e^	477 (6.5)	624 (10.3)	906 (13.8)	2415 (20.8)	1404 (15.3)	< 0.001
Unemployed in older adulthood^f^	319 (4.4)	364 (6.0)	454 (6.9)	1124 (97)	609 (6.4)	< 0.001
Sickness absence						
Long-term sickness absence in middle adulthood^g^	198 (2.7)	239 (3.9)	327 (5.0)	836 (7.2)	650 (7.1)	< 0.001
Long-term sickness absence in older adulthood^f^	393 (5.4)	416 (6.9)	563 (8.6)	1321 (11.4)	1063 (11.6)	< 0.001
Outcome^h^						
Health-related early exit	782 (10.7)	878 (14.5)	1213 (18.4)	2697 (23.2)	2256 (24.6)	< 0.001
Long-term unemployment	537 (7.3)	734 (12.1)	871 (13.2)	1862 (16.0)	1351 (14.7)	< 0.001
Early old- age pension with income	993 (13.6)	924 (15.2)	1011 (15.4)	1884 (16.2)	1489 (16.2)	< 0.001
Early old- age pension without income	1454 (19.9)	1627 (26.8)	1878 (28.5)	3395 (29.2)	2700 (29.4)	< 0.001

*SEP* Socioeconomic position, *BMI* Body mass index

^a^Measured in 1960

^b^Measured during conscription in 1969

^c^Measured from 1971 to 2003/2004/2005

^d^Measured from 1974 to 1991

^e^Measured from 1992 to1998/1999/2000

^f^Measured from 1999/2000/2001 to 2003/2004/2005

^g^ Measured from 1994 to1998/1999/2000

^h^Measured from the age of 60 until 64 years old

During follow-up, a total of 7826 (19.2%) men had left the labor force early due to health-related factors (long-term sickness absence or granted disability pension), 5355 (13.1%) men due to long-term unemployment, 6284 (15.6%) men due to early old-age pension with income and 10 963 (27.4%) men due to an early old-age pension without income (Table 1). The results of Table 2 demonstrate that almost all potential explanatory factors included in the current analyses were positively associated with the four different outcomes. Some factors measured during conscription, early health problems, and early labor market marginalization with early old-aged pension with and without income (Table 2).

**Table 2:** Unadjusted HRs and 95% CIs on the association between each potential risk factor and each Early Exit pathway separately

	Health- related early exit	Long-term unemployment	Early old-age pension with income	Early old-age pension without income
Childhood SEP				
Unskilled worker	1.82 (1.61, 2.06)	1.53 (1.32, 1.76)	1.09 (0.97, 1.23)	1.10 (1.00, 1.20)
Skilled worker	1.75 (1.54, 1.99)	1.50 (1.29, 1.74)	1.05 (0.93, 1.20)	1.12 (1.02, 1.23)
Low-level non-manual employee	1.42 (1.23, 1.63)	1.38 (1.18, 1.63)	1.02 (0.90, 1.16)	1.14 (1.02, 1.25)
Intermediate non-manual employee	1.30 (1.14, 1.48)	1.34 (1.15, 1.56)	0.99 (0.87, 1.12)	1.07 (0.97, 1.18)
High-level non-manual employee (ref)	1.00	1.00	1.00	1.00
Farmer	1.54 (1.35, 1.77)	1.07 (0.91, 1.26)	0.84 (0.78, 0.00)	1.07 (0.96, 1.18)
IQ				
High (7–9) (ref)	1.00	1.00	1.00	1.00
Medium (4–6)	1.50 (1.42, 1.58)	1.33 (1.25, 1.42)	1.10 (1.04, 1.17)	1.10 (1.05, 1.15)
Low (1–3)	2.00 (1.87, 2.13)	1.77 (1.64, 1.91)	1.12 (1.03, 1.20)	1.18 (1.11, 1.24)
Health behaviors				
Smoking	1.48 (1.42, 1.55)	1.38 (1.31, 1.46)	1.12 (1.07, 1.18)	1.11 (1.07, 1.16)
Risky use of alcohol	1.34 (1.27, 1.41)	1.26 (1.18, 1.35)	1.07 (1.01, 1.14)	1.04 (1.00, 1.09)
BMI ≥ 25	1.48 (1.37, 1.61)	1.12 (1.01, 1.25)	1.06 (0.96, 1.18)	1.00 (0.93, 1.10)
Low emotional control	1.36 (1.30, 1.43)	1.33 (1.25, 1.41)	0.97 (0.91, 1.02)	1.01 (0.97, 1.05)
Psychiatric diagnosis	1.48 (1.38, 1.58)	1.45 (1.34, 1.57)	0.95 (0.87, 1.04)	0.97 (0.91, 1.03)
Musculoskeletal diagnosis	1.19 (1.12, 1.25)	1.05 (0.98, 1.13)	0.99 (0.92, 1.05)	1.01 (0.96, 1.06)
Inpatient-care psychiatric diagnosis	2.45 (2.64, 2.65)	1.75 (1.57, 1.95)	0.98 (0.86, 1.11)	1.01 (0.92, 1.12)
Employment histories				
Youth unemployment	1.45 (1.36, 1.54)	1.42 (1.32, 1.54)	1.07 (0.99, 1.16)	1.04 (0.98, 1.10)
Unemployed in young adulthood	1.69 (1.55, 1.83)	2.20 (2.01, 2.40)	1.01 (0.91, 1.13)	1.00 (0.92, 1.09)
Unemployed in middle adulthood	1.68 (1.59, 1.77)	3.33 (3.14, 3.52)	0.93 (0.86, 1.00)	1.10 (1.05, 1.16)
Unemployed in older adulthood	1.62 (1.51, 1.74)	6.29 (5.91, 6.69)	0.81 (0.73, 0.90)	1.31 (1.22, 1.40)
Sickness absence				
Long-term sickness absence in middle adulthood	2.97 (2.78, 3.18)	1.63 (1.49, 1.80)	1.00 (0.89, 1.12)	1.10 (1.01, 1.20)
Long-term sickness absence in older adulthood	5.38 (5.12, 5.66)	1.80 (1.66, 1.94)	1.07 (0.98, 1.17)	1.20 (1.12, 1.28)

*SEP* Socio-economic position, *BMI* Body mass index

### Health-related early exit

Evidence of a graded association between years of education and health-related early exit was found (Table 3). In the crude model, men with lower levels of education had almost a 2.5-fold increased risk of being either on sickness absence or granted disability pension compared to men with the highest level of education. Concerning the contribution of labor market marginalization, including four measures of unemployment explained a considerable proportion of the education and health-related early exit association. Among the low-educated men, the hazard ratio attenuated by 11%. Including two measures of long-term sickness absence appeared to explain a larger proportion of the education and health-related early exit association compared to unemployment. Especially in older adulthood when the men were between the age of 50 and 54 years old, as the risk estimates were reduced by 14–23%. All together the risk factors attenuated the association between education and health-related early exit by 38–55%.

**Table 3:** Crude and adjusted hazard ratios (HRs) with 95% confidence intervals (CIs) for the association between level of education (years) and various early exit pathways

	≥ 15	13–14		12		10–11		≤ 9	
	HR (95%CI)	HR (95%CI)	% Δ	HR (95%CI)	% Δ	HR (95%CI)	% Δ	HR (95%CI)	% Δ
**Health-related early exit (7826 events)**									
Crude	1.00 (ref)	1.38 (1.25, 1.52)		1.79 (1.64, 1.96)		2.32 (2.14, 2.51)		2.48 (2.29, 2.69)	
Unemployment									
Before 18	1.00	1.37 (1.25, 1.51)	1	1.79 (1.63, 1.96)	2	2.25 (2.08, 2.44)	4	2.45 (2.26, 2.66)	6
Young adulthood	1.00	1.37 (1.25, 1.51)	1	1.79 (1.63, 1.96)	1	2.25 (2.08, 2.44)	5	2.46 (2.26, 2.66)	2
Middle adulthood	1.00	1.35 (1.23, 1.49)	6	1.73 (1.58, 1.90)	7	2.17 (2.00, 2.35)	11	2.38 (2.20, 2.59)	7
Older adulthood	1.00	1.37 (1.24, 1.51)	3	1.77 (1.62, 1.94)	3	2.26 (2.09, 2.45)	4	2.46 (2.27, 2.67)	2
Adjusted for all UE	1.00	1.35 (1.22, 1.49)	8	1.73 (1.58, 1.89)	8	2.10 (1.94, 2.27)	17	2.32 (2.15, 2.52)	11
Sickness absence									
Middle adulthood	1.00	1.36 (1.23, 1.49)	6	1.74 (1.59, 1.90)	7	2.19 (2.02, 2.37)	10	2.34 (2.16, 2.54)	10
Older adulthood	1.00	1.32 (1.20, 1.46)	14	1.66 (1.52, 1.82)	17	2.02 (1.86, 2.18)	23	2.16 (1.99, 2.34)	22
Adjusted for all SA	1.00	1.13 (1.19, 1.44)	18	1.64 (1.50, 1.80)	19	1.97 (1.82, 2.13)	26	2.11 (1.94, 2.29)	25
Full model	1.00	1.23 (1.12, 1.36)	38	1.47 (1.34, 1.61)	41	1.60 (1.46, 1.74)	55	1.68 (1.53, 1.84)	54
**Long-term unemployment (5355 events)**									
Crude	1.00	1.68 (1.51, 1.88)		1.86 (1.67, 2.07)		2.29 (2.08, 2.52)		2.09 (1.89, 2.31)	
Unemployment									
Before 18	1.00	1.68 (1.50, 1.87)	1	1.84 (1.65, 2.05)	2	2.22 (2.02, 2.45)	5	2.00 (1.81, 2.22)	8
Young adulthood	1.00	1.67 (1.49, 1.86)	2	1.84 (1.65, 2.05)	2	2.18 (1.98, 2.40)	9	2.05 (1.86, 2.27)	4
Middle adulthood	1.00	1.58 (1.42, 177)	14	1.65 (1.48, 1.84)	24	1.85 (1.67, 2.03)	34	1.81 (1.64, 2.00)	26
Older adulthood	1.00	1.60 (1.43, 1.79)	12	1.71 (1.54, 1.90)	17	1.95 (1.77, 2.15)	26	1.95 (1.76, 2.15)	13
Adjusted for all UE	1.00	1.55 (1.39, 1.73)	19	1.62 (1.45, 1.81)	28	1.73 (1.57, 1.90)	44	1.80 (1.63, 1.99)	27
Sickness absence									
Middle adulthood	1.00	1.67 (1.50, 1.87)	1	1.84 (1.65, 2.05)	2	2.24 (2.04, 2.47)	4	2.05 (1.85, 2.26)	4
Older adulthood	1.00	1.67 (1.49, 1.86)	2	1.82 (1.63, 2.03)	4	2.21 (2.01, 2.43)	6	2.01 (1.82, 2.22)	7
Adjusted for all SA	1.00	1.66 (1.49, 1.86)	3	1.81 (1.63, 2.02)	5	2.18 (1.94, 2.41)	8	1.99 (1.80, 2.20)	9
Full model	1.00	1.53 (1.37, 1.72)	22	1.57 (1.40, 1.75)	34	1.61 (1.45, 1.79)	52	1.66 (1.48, 1.86)	40
**Early old-age pension with income (6248 events)**									
Crude	1.00	1.14 (1.05, 1.25)		1.15 (1.06, 1.26)		1.23 (1.13, 1.33)		1.25 (1.15, 1.35)	
Unemployment									
Before 18	1.00	1.14 (1.05, 1.25)	0	1.15 (1.05, 1.26)	1	1.22 (1.13, 1.32)	2	1.25 (1.15, 1.35)	2
Young adulthood	1.00	1.14 (1.04, 1.25)	0	1.15 (1.06, 1.26)	0	1.23 (1.14, 1.33)	0	1.24 (1.15, 1.35)	0
Middle adulthood	1.00	1.15 (1.05, 1.26)	+ 3	1.15 (1.05, 1.26)	+ 5	1.23 (1.14, 1.32)	+ 7	1.24 (1.14, 1.34)	+ 4
Older adulthood	1.00	1.15 (1.05, 1.26)	+ 4	1.16 (1.06, 1.27)	+ 7	1.25 (1.16, 1.35)	+ 9	1.26 (1.16, 1.37)	+ 5
Adjusted for all UE	1.00	1.15 (1.05, 1.26)	+ 5	1.17 (1.07, 1.27)	+ 8	1.25 (1.16, 1.36)	+ 9	1.26 (1.16, 1.36)	+ 4
Sickness absence									
Middle adulthood	1.00	1.15 (1.05, 1.25)	0	1.15 (1.06, 1.26)	0	1.24 (1.14, 1.33)	+ 1	1.25 (1.15, 1.35)	0
Older adulthood	1.00	1.15 (1.05, 1.25)	0	1.16 (1.06, 1.26)	+ 1	1.24 (1.14, 1.34)	+ 1	1.25 (1.15, 1.36)	+ 1
Adjusted for all SA	1.00	1.15 (1.05, 1.25)	+ 1	1.16 (1.06, 1.26)	+ 1	1.24 (1.15, 1.34)	+ 2	1.25 (1.16, 1.36)	+ 2
Full model	1.00	1.15 (1.05, 1.26)	+ 3	1.15 (1.05, 1.26)	5	1.22 (1.12, 1.33)	4	1.23 (1.12, 1.36)	6
**Early old-age pension without income (10 963 events)**									
Crude	1.00	1.38 (1.29, 1.48)		1.50 (1.40, 1.61)		1.55 (1.46, 1.65)		1.58 (1.48, 1.69)	
Unemployment									
Before 18	1.00	1.38 (1.29, 1.49)	0	1.50 (1.40, 1.60)	0	1.56 (1.46, 1.68)	+ 1	1.59 (1.49, 1.70)	+ 2
Young adulthood	1.00	1.38 (1.29, 1.49)	0	1.50 (1.40, 1.61)	0	1.55 (1.46, 1.65)	+ 1	1.58 (1.48, 1.69)	0
Middle adulthood	1.00	1.38 (1.29, 1.48)	1	1.50 (1.39, 1.60)	1	1.54 (1.45, 1.64)	2	1.57 (1.48, 1.68)	1
Older adulthood	1.00	1.37 (1.27, 1.47)	4	1.47 (1.37, 1.57)	6	1.50 (1.41, 1.59)	9	1.55 (1.45, 1.65)	5
Adjusted for all UE	1.00	1.37 (1.28, 1.47)	3	1.48 (1.38, 1.58)	5	1.52 (1.43, 1.62)	4	1.57 (1.47, 1.67)	2
Sickness absence									
Middle adulthood	1.00	1.38 (1.29, 1.48)	0	1.50 (1.40, 1.61)	0	1.55 (1.45, 1.65)	1	1.58 (1.48, 1.68)	1
Older adulthood	1.00	1.38 (1.29, 1.48)	1	1.48 (1.39, 1.60)	3	1.53 (1.43, 1.63)	4	1.56 (1.46, 1.66)	4
Adjusted for all SA	1.00	1.38 (1.29, 1.48)	1	1.49 (1.39, 1.60)	3	1.53 (1.44, 1.63)	4	1.56 (1.46, 1.66)	4
Full model	1.00	1.39 (1.29, 1.49)	+ 2	1.50 (1.40, 1.61)	0	1.56 (1.46, 1.68)	+ 3	1.62 (1.51, 1.75)	+ 8

*HR* Hazard ratio, *Δ attenuation* Representing the proportion of the education–early exit association is explained by the risk factor in question, *UE* Unemployment, *SA* Sickness absence

Full model: Adjusted for childhood SEP, cognitive ability, smoking, alcohol, BMI, low emotional control, mental and physical health, labor market marginalization across the working life

### Long-term unemployment

Men with lower levels of education had more than a twofold increased risk of leaving the labor market early due to long-term unemployment compared to men with the highest level of education (Table 3). Previous unemployment seems to be important in explaining the association between education and early exit due to unemployment, as the risk estimates attenuated by 19–44%. When examining the measures of unemployment individually, the largest attenuation of the risk estimates was found after including a measure of unemployment in middle adulthood. Differences in long-term sickness absence during the working life explained much less of the educational differences in the outcome as the risk estimates attenuated only slightly (3–9%). After including all risk factors into the model, the graded association between education and early exit due to long-term unemployment diminished, as all men with lower levels of education had around a 1.5-fold increased risk compared to the men with the highest level of education.

### Early old-age pension with income

As seen in Table 3, men with lower levels of education had a slightly higher risk of leaving the labor market early with an old-age pension with income compared to men with the highest level of education. The various measures of labor market marginalization had very little effect on the risk estimates, which was also found in the last model when all risk factors were included in the model.

### Early old-age pension without income

Similar results were found for the outcome of early old-age pension without income (Table 3). Men with lower levels of education had more than a 1.5-fold increased risk compared to men with the highest level of education. Generally, risk factors measured across the life course had little or no effect on the risk estimates.

### Additional analyses

Results of the additional analyses, when baseline characteristics were included simultaneously as the various measures of labor market marginalization, reached the same conclusions as in the main analyses (Supplementary Table 3).

## Discussion

As expected, this study showed that lower-educated men were more likely to exit the labor force early through all the different pathways compared to same-aged highly educated men. Labor market marginalization, especially in middle adulthood and the years immediately preceding the follow-up period, explained a large part of the association between education and health-related early exit and early exit due to long-term unemployment. Risk factors measured across the life course had little effect on the educational differences found in the more voluntary early exit routes, i.e. early old-age pension with and without income.

Given that the most common early exit route among lower and higher educated men was an early old-age pension, the current results strengthen the notion that changes in social systems often result in using alternative exit routes from the labor market [10, 11]. The findings of educational differences in early exit are in line with other studies that have mainly focused on the educational differences in involuntary exit routes such as disability pension and unemployment [2, 6, 17]. Unlike previous research, the current study showed that low education is also a risk factor for exiting the workforce early in a more voluntary exit route [2, 6]. Strengthening the current research field on educational differences in early old-age pension we divided up this outcome into two based on income. A recent report from Sweden found that the early old-age pensions with and without income differ substantially, whereas the group without income was the most vulnerable [41]. A finding which was reinforced by the results of the current study. Previous research suggests that early voluntary retirees are more highly educated, have a higher income, and had more likely planned for their retirement [15]. Furthermore, individuals with high levels of education may be able to leave the labor force early through a severance package or a ‘golden handshake’, which may not be visible in our registers. On the other hand, individuals with lower levels of education might not have a choice but to take out their old-age pension early due to social, health, or work-related factors [14].

These findings could have important implications for the current policies regarding extending working with regards to increasing the state pension age. In Sweden and elsewhere, the pension reform is built on a monetary incentive to continue working, such that the more years in the labor force the greater the pension benefits will be [11]. Therefore, early exit to the regular pension system by someone with a low income means very low compensation which in turn could increase the already existing social inequalities.

Given that early social exposures and transitions in and out of the labor market shape future outcomes, a life course approach is needed to gain a better understanding of the individual's retirement decisions [26]. Accordingly, extending previous research on disability pension and labor market marginalization among mature-aged workers [17, 18], we investigate the importance of factors before labor market entry in explaining the educational differences in an early exit from the labor market. In agreement with previous research, we found that differences in childhood SEP, cognitive ability, and health behaviors, explained a large part of the association between educational qualification and health-related early exit, as well as early exit due to long-term unemployment (detailed results shown in Supplementary Tables 4 and 5). Cognitive ability, which is highly correlated with education [42], explained the largest part of the educational differences in early exit among the early risk factors. A potential reason for this could be that we included several measures of poor health behaviors (i.e. smoking and risky alcohol use) during late adolescence, which might not reflect later health behaviors. Previous twin studies from Sweden have found that healthy behaviors have a stronger (inverse) association with early exit due to disability pension and unemployment, compared to moderate alcohol use and smoking [43]. Relatively, these factors explained a much smaller part of the association between educational qualification and early old-age pension with income and had a marginal effect on the risk estimates with the outcome of early old-age pension without income (detailed results shown in Supplementary Tables 6 and 7). A potential reason for this could be that the underlying mechanisms and reasons for early old-age pensions are more varied compared to more health-related exit routes. Other factors that could potentially be of importance concerning more voluntary exit routes might be workplace factors such as more flexible working hours or given the possibility of updating and acquiring new skills [44]. With regards to social factors, previous research suggests that singles tend to extend their work life to a greater extent compared to married people, and having a partner retiring predicts retirement [44].

Deviating slightly from previous research, we found that differences in health as measured by our health indicators did not explain a large part of the associations between education and any of the early exit pathways in the current study [4, 7, 13]. A potential explanation for this discrepancy is the healthy-worker selection effect, such that older workers are healthier compared to the general older population [45, 46]. In the current study, all men included were over the age of 55 years and had not been granted a disability pension. Furthermore, poor self-assessed health is a strong predictor of unemployment [46]. Given that the most disadvantaged workers with poorer health most likely had already left the labor market the differences in health were more evenly distributed between the educational groups in the study population compared to in the general population. Men excluded from the analytical sample had much more mental and physical health problems, as well as previous unemployment and sickness absence compared to the men included, both in late adolescence and later in life. Another reason could be how health was defined and health data were collected. Previous research has to a great extent relied on self-report health measures [4, 24], whilst the current study relied on the diagnosis given by the physician and psychiatrist during conscription examination at age of 18. Furthermore, the second source of heath information came from the inpatient care register which only included the individuals with severe mental health problems. Evidence suggests that self-perceived poor health has a stronger association with an exit from paid employment compared to mental health and chronic diseases [4]. In agreement with this, and similar to previous research [4], we did not find a link between mental health and more voluntary early exit from the labor market. We did however find that sickness absence explained a large part of the association between education and health-related early exit. Although sickness absence cannot be interpreted as a direct indicator of ill-health it can be seen as a proxy measure of health and not only as a dimension of labor market marginalization [47].

Furthering previous research on educational differences in labor market marginalization among mature age workers [18], we found that unemployment, especially among older workers was a strong predictor for leaving the labor force early and explained a large part of the association between education and early exit due to long-term unemployment. Being marginalized from the labor force is associated with less favorable labor market positions characterized by lower salaries, recurring unemployment, and early exit from the labor market, especially among low-educated workers [18, 25, 48]. Results of this study highlight the importance of maintaining the older workers in the workforce by promoting older workers’ employability and creating environments supporting older workers’ retention in employment already by the age of 50 years [3, 6].

Sickness absence is also an indicator of labor market marginalization. In the current study, we included information on long-term sickness absence during two time periods in the men’s working life: middle and older adulthood. Having been on long-term sickness absence, especially in older adulthood, explained a large part of the association between education and health-related early exit but had very little effect on the other exit routes. This strengthens previous research suggesting that sickness absence, especially close to statutory retirement age, is a strong predictor of leaving the labor force early due to health reasons (16, 25–27). Given the stricter eligibility criteria for sickness absence and disability pension, there is a risk that individuals are working despite the presence of disease which could differ depending on educational qualification.

### Strengthens and limitations

The strengths of this study are the large cohort, the long-term follow-up and the use of registered data as a source of information as this provides reliable and objective information. Another strength is the ability to distinguish between the different exit routes since educational differences and underlying causes of these differences differ depending on the kind of early exit route. A limitation could be that the outcome measures were treated as a stable and straightforward event which is not always the case. For example, individuals granted disability pension often have one or more previous episodes of sickness absence [27–29]. Therefore, individuals in the current study could potentially have passed through several of the exit routes before a permanent exit was obtained. To decrease this issue, the outcomes of disability pension and long-term sickness absence were merged into one as they are so closely related. Furthermore, due to the complexity when leaving the labor force, it is difficult to define the outcome and to be certain the exit is permanent. In the current study, we could follow the individuals up to the age of 65 years and found that only 30% returned to the workforce after being censored in our analyses suggesting that we captured permanent exit to a large extent (data not shown).

An obvious limitation is that the conscription cohort only includes men. Previous research suggests that generally, the educational differences in early exit are stronger for men compared to women [6]. Given the gendered nature of the labor market and retirement timing, it would be of interest to examine if the risk factors of early exit might act differently for women. For example, previous research suggests that previous spells of unemployment are an important risk factor for early exit among men but not women [49]. Furthermore, female careers are more often interrupted by caregiving and family responsibility which could also influence retirement decisions [2]. Also, disability pension and sickness absence are more common among women compared to men [50].

In the current study, we directed our attention to labor market marginalization across the life course, an important dimension when studying early retirement, but it does not give the complete picture. The results of this study strengthen and contribute with additional evidence suggesting that an unequal distribution of unemployment and sickness absence across the working life can explain a large part of the educational differences in an early exit, at least for the involuntary exit routes. However, earlier experiences in other domains, like social factors, work characteristics, and job demand, need to be further incorporated to get a better understanding of the factors that influence early retirement. This is especially important concerning the more voluntary exit routes, such as an early old-age pension, as this route appears to be the most common early exit route today.

## Conclusion

The results of this study suggest that lower-educated men were more likely to exit the labor force early through all the different exit pathways compared to highly educated men. In the initial analyses, the strongest associations were found among early exit due to health-related reasons and unemployment and, to a lesser extent early old-age pension with and without income. Exposure to labor market marginalization across the working life was important in explaining educational differences in early labor market exit due to disability pension or sickness absence and unemployment. Labor marker marginalization had little effect on the educational differences found in the more voluntary early exit routes, i.e. early old-age pension with and without income. Although social insurance systems differ between countries the current study highlights the importance of early factors in prolonging working life which is the main objective of many pension reforms. This study underscores the importance of active labor market policy for older people, as well as identifying and implementing preventive measures in the workplace (e.g. adaptions) to prevent new spells of sickness absence and unemployment, especially among low educated individuals.

## Supplementary Information

**Additional file 1.**

## Data Availability

The data that support the findings of this study are available from Statistics Sweden but restrictions apply to the availability of these data, which were used under license for the current study, and so are not publicly available. Data are however available from the authors upon reasonable request and with permission of Statistics Sweden.

## Abbreviations

LISA: Longitudinal Register of Education and Labour Market Statistics
SEP: Socio-economic position
BMI: Body mass index
