# Supplementary material for: Educational qualification differences and early labor market exit among men: the contribution of labor market marginalization measured across the working life
Source: BMC Public Health. 2022 May 20;22:1015. doi: 10.1186/s12889-022-13397-1 (PMC9121573; doi:10.1186/s12889-022-13397-1)
Supplement: Supplementary file 1 — Additional file 1. [file 12889_2022_13397_MOESM1_ESM.docx]

Supplementary Table 1: Baseline characteristics of the individuals included and excluded in the study population

|  | Included  n(%) | Excluded  n(%) | p-value |
| --- | --- | --- | --- |
| Total | 40 761 (83.0) | 9851 (17.0) |  |
| Childhood SEP^a^  Unskilled worker  Skilled worker  Low-level non-manual employee  Intermediate non-manual employee  High-level non-manual employee  Farmer  Not classified | 12 218 (32.5)  8704 (21.4)  4251 (10.4)  6959 (17.1)  2161 (5.3)  4650 (11.4)  818 (2.0) | 3057 (36.5)  1810 (21.6)  736 (8.8)  1317 (15.7)  410 (4.9)  752 (9.0)  289 (3.5) | <0.001 |
| IQ^b^  High (7-9)  Medium (4-6)  Low (1-3)  Missing | 13 691 (33.6)  20 125 (49.4)  6913 (17.0)  31 (0.1) | 1866 (22.3)  4034 (48.2)  2462 (29.4)  9 (0.1) | <0.001 |
| Health behaviors^b^  Smoking ≥5 cigarettes/day  Risky use of alcohol  BMI ≥25 | 18 240 (44.8)  8147 (20.0)  2506 (6.2) | 4710 (56.3)  2337 (27.9)  722 (8.6) | <0.001  <0.001  <0.001 |
| Low emotional control^b^ | 11 321 (27.8) | 3549 (42.4) | <0.001 |
| Psychiatric diagnosis^b^ | 4077 (10.0) | 1840 (22.0) | <0.001 |
| Musculoskeletal diagnosis^b^ | 6706 (16.5) | 1593 (19.0) | <0.001 |
| Inpatient-care psychiatric diagnosis^c^ | 1750 (4.3) | 2176 (26.0) | <0.001 |
| Employment histories  Youth unemployment^b^  Unemployed in young adulthood^d^  Unemployed in middle adulthood^e^  Unemployed in older adulthood^f^ | 4468 (11.0)  2169 (5.3)  5826 (14.3)  2870 (7.0) | 1668 (19.9)  874 (10.4)  1743 (20.8)  354 (4.2) | <0.001  <0.001  <0.001  <0.001 |
| Sickness absence  Long-term sickness absence in middle adulthood^g^  Long-term sickness absence in older adulthood^f^ | 876 (2.2)  3756 (9.2) | 2128 (25.4)  2470 (29.5) | <0.001 |
| Years of education  ≤9  10-11  12  13-14  ≥15  Missing | 9090 (22.3)  11 657 (28.6)  6532 (16.0)  6070 (14.9)  7412 (18.2)  0 | 2267 (27.1)  2473 (29.5)  917 (11.0)  578 (6.9)  639 (7.6)  1497 (17.9) | <0.001 |

SEP: socioeconomic position, BMI: Body mass index

^a^Measured in 1960

^b^Measured during conscription in 1969

^c^Measured from 1971 to 2003/2004/2005

^d^Measured from 1974 to 1991

^e^Measured from 1992 to1998/1999/2000

^f^Measured from 1999/2000/2001 to 2003/2004/2005

^g^ Measured from 1994 to1998/1999/2000

Supplementary Table 2. Complete case analyses excluding 1752 individuals with missing information on potential explanatory factors, crude and adjusted hazard ratios (HRs) with 95% confidence intervals (CIs) for the association between level of education (years) and various early exit pathways.

|  | ≥15 | 13-14 |  | 12 |  | 10-11 |  | ≤9 |  |
| --- | --- | --- | --- | --- | --- | --- | --- | --- | --- |
|  | HR (95%CI) | HR (95%CI) | % Δ | HR (95%CI) | % Δ | HR (95%CI) | % Δ | HR (95%CI) | % Δ |
| **Sickness absence or disability pension (7496 events)** | | | | | | | | | |
| Crude | 1.00 | 1.39 (1.26, 1.53) |  | 1.78 (1.62, 1.95) |  | 2.30 (2.12, 2.50) |  | 2.49 (2.29, 2.70) |  |
| Unemployment | 1.00 | 1.36 (1.23, 1.50) | 8 | 1.71 (1.56, 1.88) | 8 | 2.09 (1.92, 2.27) | 16 | 2.33 (2.14, 2.54) | 10 |
| Sickness absence | 1.00 | 1.33 (1.20, 1.46) | 16 | 1.63 (1.49, 1.79) | 19 | 1.97 (1.81, 2.13) | 26 | 2.11 (1.94, 2.29) | 26 |
| Full model | 1.00 | 1.25 (1.13, 1.38) | 36 | 1.45 (1.32, 1.60) | 42 | 1.59 (1.45, 1.74) | 55 | 1.67 (1.52, 1.84) | 55 |
|  |  |  |  |  |  |  |  |  |  |
| **Unemployment (5106 events)** | | | | | | | | | |
| Crude | 1.00 | 1.76 (1.56, 1.97) |  | 1.92 (1.72, 2.14) |  | 2.33 (2.11, 2.58) |  | 2.14 (1.93, 2.37) |  |
| Unemployment | 1.00 | 1.62 (1.44, 1.81) | 18 | 1.67 (1.50, 1.87) | 26 | 1.77 (1.60, 1.96) | 42 | 1.85 (1.66, 2.05) | 26 |
| Sickness absence | 1.00 | 1.74 (1.55, 1.95) | 3 | 1.87 (1.67, 2.09) | 5 | 2.23 (2.01, 2.46) | 8 | 2.03 (1.83, 2.25) | 9 |
| Full model | 1.00 | 1.60 (1.43, 1.80) | 21 | 1.61 (1.44, 1.81) | 33 | 1.64 (1.47, 1.83) | 52 | 1.69 (1.50, 1.90) | 40 |
|  |  |  |  |  |  |  |  |  |  |
| **Old age pension with income (6025 events)** | | | | | | | | | |
| Crude | 1.00 | 1.14 (1.04, 1.25) |  | 1.14 (1.04, 1.25) |  | 1.22 (1.12, 1.32) |  | 1.25 (1.15, 1.36) |  |
| Unemployment | 1.00 | 1.15 (1.05, 1.26) | +5 | 1.15 (1.05, 1.26) | +9 | 1.24 (1.14, 1.34) | +10 | 1.26 (1.16, 1.37) | +4 |
| Sickness absence | 1.00 | 1.14 (1.04, 1.25) | +1 | 1.14 (1.05, 1.25) | +2 | 1.22 (1.13, 1.32) | +2 | 1.26 (1.16, 1.36) | +2 |
| Full model | 1.00 | 1.15 (1.05, 1.27) | +7 | 1.14 (1.04, 1.25) | 2 | 1.21 (1.11, 1.32) | 3 | 1.24 (1.13, 1.36) | 4 |
|  |  |  |  |  |  |  |  |  |  |
| **Old age pension without income (10 506 events)** | | | | | | | | | |
| Crude | 1.00 | 1.38 (1.28, 1.48) |  | 1.51 (1.41, 1.62) |  | 1.54 (1.45, 1.64) |  | 1.58 (1.48, 1.69) |  |
| Unemployment | 1.00 | 1.37 (1.27, 1.47) | 3 | 1.49 (1.38, 1.59) | 4 | 1.52 (1.43, 1.62) | 4 | 1.57 (1.47, 1.68) | 1 |
| Sickness absence | 1.00 | 1.38 (1.28, 1.48) | 1 | 1.49 (1.39, 1.60) | 2 | 1.52 (1.43, 1.62) | 4 | 1.56 (1.46, 1.66) | 4 |
| Full model | 1.00 | 1.39 (1.29, 1.49) | +2 | 1.51 (1.41, 1.62) | +1 | 1.57 (1.46, 1.68) | +4 | 1.63 (1.51, 1.76) | +9 |

HR Hazard ratio; Δ attenuation, representing the proportion of the education–early exit association is explained by the risk factor in question.

Crude: no adjustments

Unemployment: unemployed before 18, unemployed in young adulthood, unemployed in middle adulthood, unemployed in older adulthood

Sickness absence: sickness absence during middle adulthood and sickness absence in older adulthood

Full model: Adjusted for childhood SEP, cognitive ability, smoking, alcohol, BMI, low emotional control, mental and physical health, labor market marginalization across the working life

Supplementary Table 3. Crude and adjusted hazard ratios (HRs) with 95% confidence intervals (CIs) for the association between level of education (years) and various early exit pathways.

|  | ≥15 | 13-14 |  | 12 |  | 10-11 |  | ≤9 |  |
| --- | --- | --- | --- | --- | --- | --- | --- | --- | --- |
|  | HR (95%CI) | HR (95%CI) | % Δ | HR (95%CI) | % Δ | HR (95%CI) | % Δ | HR (95%CI) | % Δ |
| **Health related early exit (7826 events)** | | | | | | | | | |
| Model 1 | 1.00 | 1.29 (1.17, 1.42) |  | 1.57 (1.43, 1.72) |  | 1.81 (1.66, 1.98) |  | 1.83 (1.67, 2.01) |  |
| Model 1 + Unemployment | 1.00 | 1.27 (1.16, 1.40) | 5 | 1.55 (1.41, 1.69) | 4 | 1.73 (1.58, 1.89) | 10 | 1.82 (1.66, 1.99) | 1 |
| Model 1 + Sickness absence | 1.00 | 1.24 (1.13, 1.37) | 15 | 1.48 (1.34, 1.62) | 16 | 1.64 (1.52, 1.80) | 21 | 1.69 (1.54, 1.86) | 17 |
| Full model | 1.00 | 1.23 (1.12, 1.36) | 18 | 1.47 (1.34, 1.61) | 18 | 1.60 (1.46, 1.74) | 26 | 1.68 (1.53, 1.84) | 18 |
| **Unemployment (5355 events)** | | | | | | | | | |
| Model 1 | 1.00 | 1.65 (1.47, 1.85) |  | 1.73 (1.55, 1.94) |  | 1.97 (1.78, 2.21) |  | 1.73 (1.56, 1.94) |  |
| Model 1 + Unemployment | 1.00 | 1.54 (1.38, 1.72) | 16 | 1.58 (1.41, 1.76) | 21 | 1.63 (1.47, 1.81) | 35 | 1.68 (1.50, 1.88) | 7 |
| Model 1+ Sickness absence | 1.00 | 1.64 (1.46, 1.83) | 2 | 1.70 (1.53, 1.90) | 3 | 1.93 (1.73, 2.14) | 5 | 1.70 (1.51, 1.90) | 5 |
| Full model | 1.00 | 1.53 (1.37, 1.72) | 17 | 1.57 (1.40, 1.75) | 22 | 1.61 (1.45, 1.79) | 37 | 1.66 (1.48, 1.86) | 10 |
| **Old age pension with income (6248 events)** | | | | | | | | | |
| Model 1 | 1.00 | 1.14 (1.04, 1.25) |  | 1.13 (1.03, 1.24) |  | 1.20 (1.10, 1.31) |  | 1.23 (1.12, 1.35) |  |
| Model 1+ Unemployment | 1.00 | 1.15 (1.05, 1.26) | +6 | 1.15 (1.05, 1.26) | +9 | 1.22 (1.12, 1.33) | +10 | 1.23 (1.12, 1.35) | 0 |
| Model 1 + Sickness absence | 1.00 | 1.14 (1.04, 1.25) | +1 | 1.13 (1.04, 1.24) | +1 | 1.21 (1.11, 1.31) | 0 | 1.23 (1.12, 1.35) | 0 |
| Full model | 1.00 | 1.15 (1.05, 1.26) | +6 | 1.15 (1.05, 1.26) | +9 | 1.22 (1.12, 1.33) | +10 | 1.23 (1.12, 1.36) | 0 |
| **Old age pension without income (10 963 events)** | | | | | | | | | |
| Model 1 | 1.00 | 1.41 (1.31, 1.51) |  | 1.53 (1.43, 1.64) |  | 1.60 (1.49, 1.71) |  | 1.64 (1.53, 1.77) |  |
| Model 1 + Unemployment | 1.00 | 1.39 (1.30, 1.50) | 3 | 1.51 (1.40, 1.62) | 5 | 1.57 (1.47, 1.68) | 5 | 1.63 (1.52, 1.76) | 2 |
| Model 1 + Sickness absence | 1.00 | 1.40 (1.31, 1.51) | 1 | 1.52 (1.42, 1.64) | 2 | 1.59 (1.48, 1.70) | 2 | 1.63 (1.52, 1.76) | 2 |
| Full model | 1.00 | 1.39 (1.29, 1.49) | 4 | 1.50 (1.40, 1.61) | 6 | 1.56 (1.46, 1.68) | 6 | 1.62 (1.51, 1.75) | 3 |

HR, Hazard ratio; Δ attenuation, representing the proportion of the education–early exit association is explained by the risk factor in question.

Model 1: Adjusted for childhood SEP, cognitive ability, smoking, alcohol, BMI, low emotional control, mental and physical health

Model 1 and unemployment: Additional adjustment for unemployment across the working life

Model 1 and sickness absence: Additional adjustment for sickness absence across the working life

Full model: Adjusted for childhood SEP, cognitive ability, smoking, alcohol, BMI, low emotional control, mental and physical health, labor market marginalization across the working life

Supplementary Table 4. Detailed account of the crude and adjusted hazard ratios (HRs) with 95% confidence intervals (CIs) for the association between level of education (years) and health-related early exit.

| Adjustments | ≥15  HR (95%CI) | 13-14  HR (95%CI) | 12  HR (95%CI) | 10-11  HR (95%CI) | ≤9  HR (95%CI) |
| --- | --- | --- | --- | --- | --- |
| **Crude** | 1.00 | 1.38 (1.25, 1.52) | 1.79 (1.64, 1.96) | 2.32 (2.14, 2.51) | 2.48 (2.29, 2.70) |
| Childhood SEP at age 9–11 | 1.00 | 1.34 (1.22, 1.48) | 1.73 (1.58, 1.90) | 2.20 (2.03, 2.39) | 2.36 (2.17, 2.57) |
| IQ at age 18/19 | 1.00 | 1.34 (1.22, 1.48) | 1.67 (1.53, 1.84) | 2.04 (1.88, 2.22) | 2.10 (1.92, 2.30) |
| Health behaviors at age 18/19 | 1.00 | 1.35 (1.22, 1.48) | 1.70 (1.55, 1.86) | 2.13 (1.96, 2.30) | 2.24 (2.06, 2.44) |
| Low emotional control at age 18/19 | 1.00 | 1.38 (1.25, 1.52) | 1.79 (1.64, 1.96) | 2.28 (2.11, 2.47) | 2.42 (2.23, 2.63) |
| **Adjusted for all above** | 1.00 | 1.29 (1.17, 1.42) | 1.57 (1.43, 1.72) | 1.84 (1.68, 2.01) | 1.86 (1.69, 2.04) |
| **% reduction of HR** |  | 23 % | 28 % | 36 % | 42 % |
| Psychiatric diagnosis at age 18/19 | 1.00 | 1.38 (1.26, 1.52) | 1.79 (1.64, 1.96) | 2.29 (2.11, 2.48) | 2.44 (2.25, 2.64) |
| Musculoskeletal diagnosis at age 18/19 | 1.00 | 1.38 (1.25, 1.52) | 1.80 (1.64, 1.97) | 2.32 (2.14, 2.51) | 2.48 (2.29, 2.69) |
| Mental diagnosis (1971–2003/2005) | 1.00 | 1.37 (1.24, 1.51) | 1.77 (1.62, 1.94) | 2.25 (2.08, 2.43) | 2.40 (2.21, 2.61) |
| **Adjusted for all health-related variables** | 1.00 | 1.37 (1.25, 1.51) | 1.78 (1.62, 1.94) | 2.23 (2.06, 2.42) | 2.36 (2.18, 2.56) |
| **% reduction of HR** |  | 2 % | 2% | 6% | 8 % |
| Youth unemployment (before 18 years) | 1.00 | 1.38 (1.25, 1.51) | 1.78 (1.63, 1.95) | 2.26 (2.08, 2.45) | 2.40 (2.21, 2.61) |
| Unemployed in young adulthood  (between the age of 20 and 39-41 years old) | 1.00 | 1.37 (1.25, 1.51) | 1.79 (1.62, 1.96) | 2.25 (2.08, 2.44) | 2.46 (2.26, 2.66) |
| Unemployed in middle adulthood  (between the age of 43-45 and 50 years old) | 1.00 | 1.35 (1.23, 1.49) | 1.73 (1.58, 1.90) | 2.17 (2.00, 2.35) | 2.38 (2.20, 2.59) |
| Unemployed in older adulthood  (between the age of 50 and 59 years old) | 1.00 | 1.37 (1.24, 1.51) | 1.77 (1.62, 1.94) | 2.26 (2.09, 2.45) | 2.46 (2.27, 2.67) |
| **Adjusted for all unemployment variables** | 1.00 | 1.35 (1.22, 1.49) | 1.72 (1.58, 1.89) | 2.10 (1.94, 2.27) | 2.32 (2.14, 2.52) |
| **% reduction of HR** |  | 8% | 8% | 17% | 11% |
| Sickness absence in middle adulthood  (between the age 42-44 and 50 years) | 1.00 | 1.36 (1.23, 1.49) | 1.74 (1.59, 1.90) | 2.19 (2.02, 2.37) | 2.34 (2.16, 2.54) |
| Sickness absence in older adulthood  (between the age of 50 and 59 years) | 1.00 | 1.32 (1.20, 1.46) | 1.66 (1.52, 1.82) | 2.02 (1.86, 2.18) | 2.16 (1.99, 2.34) |
| **Adjusted for all sickness absence variables** | 1.00 | 1.31 (1.19, 1.44) | 1.64 (1.50, 1.80) | 1.97 (1.82, 2.13) | 2.11 (1.94, 2.29) |
| **% reduction of HR** |  | 18% | 19% | 26 & | 25 % |
| Full model | 1.00 | 1.23 (1.12, 1.36) | 1.47 (1.36, 1.61) | 1.60 (1.46, 1.74) | 1.68 (1.53, 1.84) |
| **% reduction of HR** |  | 38% | 41% | 55% | 54 % |

Supplementary Table 5. Detailed account of the crude and adjusted hazard ratios (HRs) with 95% confidence intervals (CIs) for the association between level of education (years) and long-term unemployment.

| Adjustments | ≥15  HR (95%CI) | 13–14  HR (95%CI) | 12  HR (95%CI) | 10–11  HR (95%CI) | ≤9  HR (95%CI) |
| --- | --- | --- | --- | --- | --- |
| **Crude** | 1.00 | 1.68 (1.51, 1.88) | 1.86 (1.67, 2.07) | 2.29 (2.08, 2.52) | 2.09 (1.89, 2.31) |
| Childhood SEP at age 9–11 | 1.00 | 1.70 (1.52, 1.90) | 1.87 (1.68, 2.08) | 2.31 (2.10, 2.56) | 2.16 (1.94, 2.39) |
| IQ at age 18/19 | 1.00 | 1.65 (1.47, 1.84) | 1.75 (1.57, 1.96) | 2.05 (1.85, 2.27) | 1.79 (1.60, 1.99) |
| Health behaviors at age 18/19 | 1.00 | 1.65 (1.48, 1.85) | 1.78 (1.60, 1.99) | 2.14 (1.95, 2.36) | 1.94 (1.75, 2.14) |
| Low emotional control at age 18/19 | 1.00 | 1.69 (1.51, 1.89) | 1.85 (1.67, 2.07) | 2.26 (2.05, 2.49) | 2.04 (1.84, 2.25) |
| **Adjusted for all above** | 1.00 | 1.65 (1.47, 1.85) | 1.73 (1.55, 1.94) | 1.99 (1.79, 2.21) | 1.75 (1.56, 1.96) |
| **% reduction of HR** |  | 5% | 14% | 23% | 32% |
| Psychiatric diagnosis at age 18/19 | 1.00 | 1.69 (1.51, 1.89) | 1.86 (1.67, 2.07) | 2.26 (2.05, 2.49) | 2.05 (1.85, 2.26) |
| Musculoskeletal diagnosis at age 18/19 | 1.00 | 1.68 (1.51, 1.88) | 1.86 (1.67, 2.07) | 2.29 (2.08, 2.52) | 2.09 (1.89, 2.31) |
| Mental diagnosis (1971–2003/2005) | 1.00 | 1.67 (1.50, 1.87) | 1.84 (1.65, 2.05) | 2.25 (2.05, 2.48) | 2.05 (1.86, 2.27) |
| **Adjusted for all health-related variables** | 1.00 | 1.68 (1.50, 1.88) | 1.84 (1.66, 2.05) | 2.23 (2.03, 2.45) | 2.02 (1.82, 2.23) |
| **% reduction of HR** |  | 1% | 1% | 5% | 7% |
| Youth unemployment (before 18 years) | 1.00 | 1.68 (1.50, 1.87) | 1.84 (1.65, 2.05) | 2.22 (2.02, 2.45) | 2.00 (1.81, 2.22) |
| Unemployed in young adulthood  (between the age of 20 and 39-41 years old) | 1.00 | 1.68 (1.49, 1.86) | 1.84 (1.65, 2.05) | 2.18 (1.98, 2.40) | 2.05 (1.86, 2.27) |
| Unemployed in middle adulthood  (between the age of 43-45 and 50 years old) | 1.00 | 1.58 (1.42, 1.77) | 1.65 (1.49, 1.84) | 1.85 (1.67, 2.03) | 1.81 (1.64, 2.00) |
| Unemployed in older adulthood  (between the age of 50 and 59 years old) | 1.00 | 1.60 (1.43, 1.79) | 1.71 (1.54, 1.90) | 1.95 (1.77, 2.15) | 1.95 (1.76, 2.15) |
| **Adjusted for all unemployment variables** | 1.00 | 1.55 (1.39, 1.73) | 1.62 (1.45, 1.81) | 1.73 (1.57, 1.90) | 1.80 (1.63, 1.99) |
| **% reduction of HR** |  | 19% | 28% | 44% | 27% |
| Sickness absence in middle adulthood  (between the age 42-44 and 50 years) | 1.00 | 1.67 (1.50, 1.87) | 1.84 (1.65, 2.05) | 2.24 (2.04, 2.47) | 2.05 (1.85, 2.26) |
| Sickness absence in older adulthood  (between the age of 50 and 59 years) | 1.00 | 1.67 (1.49, 1.86) | 1.82 (1.63, 2.03) | 2.21 (2.01, 2.43) | 2.01 (1.82, 2.22) |
| **Adjusted for all sickness absence variables** | 1.00 | 1.66 (1.49, 1.76) | 1.81 (1.63, 2.02) | 2.18 (1.99, 2.41) | 1.99 (1.80, 2.20) |
| **% reduction of HR** |  | 3% | 5% | 8% | 9% |
| Full model | 1.00 | 1.53 (1.37, 1.72) | 1.57 (1.40, 1.75) | 1.61 (1.45, 1.79) | 1.66 (1.48, 1.86) |
| **% reduction of HR** |  | 22% | 34% | 52% | 40% |

Supplementary Table 6. Detailed account of the crude and adjusted hazard ratios (HRs) with 95% confidence intervals (CIs) for the association between level of education (years) and old age pension with income.

| Adjustments | ≥15  HR (95%CI) | 13–14  HR (95%CI) | 12  HR (95%CI) | 10–11  HR (95%CI) | ≤9  HR (95%CI) |
| --- | --- | --- | --- | --- | --- |
| **Crude** | 1.00 | 1.14 (1.04, 1.25) | 1.15 (1.06, 1.26) | 1.23 (1.14, 1.32) | 1.25 (1.15, 1.35) |
| Childhood SEP at age 9-11 | 1.00 | 1.16 (1.06, 1.26) | 1.16 (1.06, 1.27) | 1.24 (1.15, 1.35) | 1.27 (1.17, 1.38) |
| IQ at age 18/19 | 1.00 | 1.14 (1.04, 1.24) | 1.14 (1.04, 1.25) | 1.21 (1.12, 1.32) | 1.23 (1.12, 1.34) |
| Health behaviors at age 18/19 | 1.00 | 1.14 (1.04, 1.24) | 1.14 (1.04, 1.24) | 1.20 (1.11, 1.30) | 1.21 (1.12, 1.31) |
| Low emotional control at age 18/19 | 1.00 | 1.14 (1.05, 1.25) | 1.15 (1.06, 1.26) | 1.24 (1.15, 1.34) | 1.25 (1.16, 1.36) |
| **Adjusted for all above** | 1.00 | 1.14 (1.04, 1.25) | 1.13 (1.03, 1.24) | 1.20 (1.10, 1.31) | 1.23 (1.12, 1.35) |
| **% reduction of HR** |  | 2% | 13% | 14% | 9% |
| Psychiatric diagnosis at age 18/19 | 1.00 | 1.14 (1.05, 1.25) | 1.15 (1.06, 1.26) | 1.24 (1.14, 1.34) | 1.25 (1.16, 1.36) |
| Musculoskeletal diagnosis at age 18/19 | 1.00 | 1.14 (1.04, 1.25) | 1.15 (1.05, 1.26) | 1.23 (1.14, 1.33) | 1.25 (1.15, 1.35) |
| Mental diagnosis (1971-2003/2005) | 1.00 | 1.15 (1.05, 1.25) | 1.15 (1.06, 1.26) | 1.23 (1.14, 1.33) | 1.25 (1.15, 1.35) |
| **Adjusted for all health-related variables** | 1.00 | 1.14 (1.05, 1.25) | 1.15 (1.06, 1.26) | 1.24 (1.15, 1.33) | 1.25 (1.16, 1.36) |
| **% reduction of HR** |  | 0% | +1% | +2% | +3% |
| Youth unemployment (before 18 years) | 1.00 | 1.14 (1.05, 1.25) | 1.15 (1.06, 1.26) | 1.23 (1.14, 1.33) | 1.24 (1.15, 1.35) |
| Unemployed in young adulthood  (between the age of 20 and 39-41 years old) | 1.00 | 1.15 (1.05, 1.25) | 1.15 (1.06, 1.26) | 1.23 (1.14, 1.33) | 1.25 (1.15, 1.35) |
| Unemployed in middle adulthood  (between the age of 43-45 and 50 years old) | 1.00 | 1.15 (1.05, 1.26) | 1.16 (1.06, 1.27) | 1.25 (1.15, 1.35) | 1.26 (1.16, 1.36) |
| Unemployed in older adulthood  (between the age of 50 and 59 years old) | 1.00 | 1.15 (1.05, 1.26) | 1.17 (1.07, 1.27) | 1.26 (1.16, 1.36) | 1.26 (1.16, 1.37) |
| **Adjusted for all unemployment variables** | 1.00 | 1.15 (1.05, 1.25) | 1.17 (1.07, 1.27) | 1.25 (1.16, 1.36) | 1.26 (1.16, 1.36) |
| **% reduction of HR** |  | +5% | +8% | +9% | +4% |
| Sickness absence in middle adulthood  (between the age 42-44 and 50 years) | 1.00 | 1.15 (1.04, 1.25) | 1.15 (1.06, 1.26) | 1.23 (1.14, 1.33) | 1.25 (1.15, 1.35) |
| Sickness absence in older adulthood  (between the age of 50 and 59 years) | 1.00 | 1.15 (1.05, 1.25) | 1.16 (1.06, 1.26) | 1.24 (1.14, 1.34) | 1.25 (1.15, 1.36) |
| **Adjusted for all sickness absence variables** | 1.00 | 1.15 (1.05, 1.25) | 1.16 (1.06, 1.26) | 1.24 (1.15, 1.34) | 1.25 (1.16, 1.36) |
| **% reduction of HR** |  | +1% | +1% | +2% | +2% |
| Full model | 1.00 | 1.15 (1.05, 1.26) | 1.15 (1.05, 1.26) | 1.22 (1.12, 1.33) | 1.23 (1.12, 1.35) |
| **% reduction of HR** |  | +3% | 5% | 5% | 6% |

Supplementary Table 7. Detailed account of the crude and adjusted hazard ratios (HRs) with 95% confidence intervals (CIs) for the association between level of education (years) and old age pension without income.

| Adjustments | ≥15  HR (95%CI) | 13–14  HR (95%CI) | 12  HR (95%CI) | 10–11  HR (95%CI) | ≤9  HR (95%CI) |
| --- | --- | --- | --- | --- | --- |
| **Crude** | 1.00 | 1.38 (1.29, 1.48) | 1.50 (1.40, 1.61) | 1.55 (1.46, 1.64) | 1.58 (1.48, 1.69) |
| Childhood SEP at age 9–11 | 1.00 | 1.41 (1.31, 1.51) | 1.53 (1.43, 1.64) | 1.60 (1.50, 1.70) | 1.63 (1.53, 1.77) |
| IQ at age 18/19 | 1.00 | 1.39 (1.29, 1.49) | 1.51 (1.41, 1.62) | 1.57 (1.47, 1.68) | 1.61 (1.50, 1.72) |
| Health behaviors at age 18/19 | 1.00 | 1.38 (1.28, 1.48) | 1.49 (1.39, 1.59) | 1.53 (1.44, 1.63) | 1.56 (1.46, 1.66) |
| Low emotional control at age 18/19 | 1.00 | 1.38 (1.29, 1.48) | 1.50 (1.40, 1.61) | 1.55 (1.46, 1.65) | 1.58 (1.48, 1.69) |
| **Adjusted for all above** | 1.00 | 1.41 (1.31, 1.51) | 1.53 (1.43, 1.65) | 1.60 (1.49, 1.71) | 1.64 (1.53, 1.77) |
| **% reduction of HR** |  | +6 % | +6 % | +9 % | +11 % |
| Psychiatric diagnosis at age 18/19 | 1.00 | 1.38 (1.29, 1.48) | 1.50 (1.40, 1.61) | 1.55 (1.46, 1.65) | 1.59 (1.49, 1.69) |
| Musculoskeletal diagnosis at age 18/19 | 1.00 | 1.38 (1.29, 1.48) | 1.50 (1.40, 1.60) | 1.55 (1.46, 1.65) | 1.58 (1.48, 1.68) |
| Mental diagnosis (1971-2003/2005) | 1.00 | 1.38 (1.29, 1.49) | 1.50 (1.40, 1.60) | 1.55 (1.45, 1.64) | 1.58 (1.48, 1.69) |
| **Adjusted for all health-related variables** | 1.00 | 1.38 (1.29, 1.48) | 1.50 (1.40, 1.60) | 1.55 (1.46, 1.65) | 1.59 (1.49, 1.69) |
| **% reduction of HR** |  | 0 % | 0 % | +1 % | +1 % |
| Youth unemployment (before 18 years) | 1.00 | 1.38 (1.29, 1.49) | 1.50 (1.40, 1.60) | 1.55 (1.46, 1.65) | 1.58 (1.48, 1.68) |
| Unemployed in young adulthood  (between the age of 20 and 39-41 years old) | 1.00 | 1.38 (1.29, 1.48) | 1.50 (1.40, 1.61) | 1.55 (1.46, 1.66) | 1.58 (1.48, 1.69) |
| Unemployed in middle adulthood  (between the age of 43-45 and 50 years old) | 1.00 | 1.38 (1.29, 1.48) | 1.50 (1.39, 1.60) | 1.54 (1.44, 1.63) | 1.57 (1.48, 1.68) |
| Unemployed in older adulthood  (between the age of 50 and 59 years old) | 1.00 | 1.37 (1.27, 1.47) | 1.47 (1.37, 1.58) | 1.50 (1.41, 1.60) | 1.55 (1.45, 1.65) |
| **Adjusted for all unemployment variables** | 1.00 | 1.37 (1.28, 1.47) | 1.48 (1.38, 1.58) | 1.52 (1.43, 1.62) | 1.57 (1.47, 1.67) |
| **% reduction of HR** |  | 3% | 5% | 5% | 2% |
| Sickness absence in middle adulthood  (between the age 42-44 and 50 years) | 1.00 | 1.38 (1.29, 1.48) | 1.50 (1.40, 1.61) | 1.55 (1.45, 1.65) | 1.58 (1.48, 1.68) |
| Sickness absence in older adulthood  (between the age of 50 and 59 years) | 1.00 | 1.38 (1.28, 1.48) | 1.49 (1.39, 1.59) | 1.53 (1.44, 1.63) | 1.56 (1.46, 1.66) |
| **Adjusted for all sickness absence variables** | 1.00 | 1.38 (1.28, 1.48) | 1.49 (1.39, 1.60) | 1.53 (1.44, 1.62) | 1.56 (1.46, 1.66) |
| **% reduction of HR** |  | 1% | 3% | 4% | 4% |
| Full model | 1.00 | 1.39 (1.29, 1.49) | 1.50 (1.40, 1.61) | 1.57 (1.46, 1.68) | 1.62 (1.50, 1.74) |
| **% reduction of HR** |  | +2 % | 0% | +3% | +8% |
